# Temporal expression and functional analysis of long non‐coding RNAs in colorectal cancer initiation

**DOI:** 10.1111/jcmm.14300

**Published:** 2019-03-28

**Authors:** Lei Dai, Junshu Li, Zhexu Dong, Yi Liu, Ye Chen, Na Chen, Lin Cheng, Chao Fang, Huiling Wang, Yanhong Ji, Shuang Chen, Xiaolan Su, Gang Shi, Yi Lin, Shuang Zhang, Yang Yang, Meng Qiu, Dechao Yu, Wei Huang, Zongguang Zhou, Yuquan Wei, Hongxin Deng

**Affiliations:** ^1^ State Key Laboratory of Biotherapy and Cancer Center West China Hospital Sichuan University and Collaborative Innovation Center for Biotherapy Chengdu P. R. China; ^2^ Department of Medical Oncology, Cancer Center the State Key Laboratory of Biotherapy West China Hospital West China Medical School Sichuan University Chengdu P. R. China; ^3^ Department of Gastrointestinal Surgery West China Hospital and State Key Laboratory of Biotherapy Sichuan University Chengdu P. R. China; ^4^ Department of Biotherapy, Cancer Center West China Hospital Sichuan University and Collaborative Innovation Center for Biotherapy Chengdu P. R. China; ^5^ West China‐Liverpool Biomedical Research Center West China Hospital/West China Medical School Sichuan University Chengdu P. R. China

**Keywords:** colorectal cancer, expression profile, H19, long noncoding RNAs, temporal expression

## Abstract

Long non‐coding RNAs (lncRNAs) have potential applications in clinical diagnosis and targeted cancer therapies. However, the expression profile of lncRNAs in colorectal cancer (CRC) initiation is still unclear. In this study, the expression profiles of lncRNAs and mRNAs were determined by microarray at specific tumour stages in an AOM/DSS‐induced primary colon cancer model. The temporal expression of lncRNAs was analysed by K‐means clustering. Additionally, weighted correlation network analysis (WGCNA) and gene ontology analysis were performed to construct co‐expression networks and establish functions of the identified lncRNAs and mRNAs. Our results suggested that 4307 lncRNAs and 5798 mRNAs are deregulated during CRC initiation. These differential expression genes (DEGs) exhibited a clear correlation with the differential stage of tumour initiation. WGCNA results suggested that a series of hub lncRNAs are involved in regulating cell stemness, colon inflammation, oxidative stress response and cell death at each stage. Among them, lncRNA H19 was up‐regulated in colon tumours and correlated with poor patient prognosis. Collectively, we have been the first to demonstrate the temporal expression and function of lncRNAs in CRC initiation. These results provide novel diagnosis and therapy targets for CRC.

## 1 INTRODUCTION

Colorectal cancer (CRC) is the third most commonly diagnosed cancer in men and the second in women worldwide, resulting in over 690 000 deaths annually.^1^ Although the survival of CRC patients has doubled in the last 40 years, the 5‐year survival rate remains low at only 65%.^2^ The overwhelming morbidity and mortality caused by CRC affects the global economy through loss of productivity and burden on the healthcare system.^3^ Various studies have demonstrated that early diagnosis and targeted therapy are efficient strategies for reducing mortality.^4^, ^5^ Consequently, researchers have expended considerable resources to understand the underlying molecular mechanisms responsible for CRC tumorigenesis and to develop novel diagnosis and therapy targets for CRC at the coding RNA, microRNAs (miRNA) and epigenetic level.^6^, ^7^

Non‐coding RNAs (ncRNAs) are RNA transcripts that do not code for proteins. Recently, numerous ncRNAs, including long ncRNAs (lncRNAs), miRNA and circular RNAs, have been discovered owing to advances in next generation RNA sequencing (RNA‐Seq) in animals. LncRNAs are more than 200 nucleotides in length and are differentially expressed in particular tissues as well as in different tumour types.^8^ LncRNAs and mRNAs share many features, as both are transcribed by RNA polymerase II (Pol II), 5ʹ‐capped, spliced, polyadenylated and are biochemically identical. However, lncRNAs usually are shorter in length, have fewer but longer exons and have lower primary sequence conservation compared with mRNAs.^9^ Although lncRNAs do not encode proteins, they still have many functions. In both the innate and adaptive immune system, lncRNAs play an important role in regulating the expression of cytokine genes and the development of immune cells, including myeloid cells, dendritic cells, T cells and B lymphocytes.^10^ The functions of lncRNAs are also crucial in both developmental and differentiation processes in mammals via controlling specific gene expressions.^11^ Moreover, lncRNAs have strong relationships with tumorigenesis. LncRNAs can influence the transcription of tumour‐related genes by altering epigenetic modifications or regulating enhancers.^12^ A few lncRNAs are revealed to act as tumour suppressors that regulate cancer signalling pathways such as p53 tumour suppressor signalling.^13^ Therefore, lncRNAs may be great biomarkers in clinical diagnosis and provide effective potential targets in future cancer therapies.

Several lncRNAs were demonstrated to be involved in regulating proliferation, migration and chemotherapy resistance and predicting prognosis of patients in colon cancer.^14^, ^15^ However, the temporal expression profile of lncRNAs in CRC initiation was still unclear. In this study, we aimed to investigate the expression and function of lncRNAs at each stage of CRC initiation. The genome‐wide annotation of temporally deregulated lncRNAs may improve our understanding of molecular mechanisms that underpin colon cancer initiation and provide a large number of candidate prognostic and therapeutic targets for CRC.

## 2 MATERIALS AND METHODS

### 2.1 Animals

C57BL/6J mice (20 g weight, male) were purchased from Beijing HFK Bioscience Co. (Beijing, China) and housed in a specific‐pathogen free room (25°C) under a 12‐h light/dark cycle with ad libitum access to food and water. All mouse care and experiments were carried out in accordance with Sichuan University guidelines concerning animal use and care.

### 2.2 Mouse model for primary colon cancer

Mice were injected intraperitoneally with 10 mg/kg azoxymethane (AOM; Sigma‐Aldrich, Germany). After 7 days, the mice received drinking water containing 2% dextran sodium sulfate (DSS; MP Biomedicals, CA, USA) for 7 days followed by normal drinking water for 14 days.^16^ Totally, three DSS cycles were performed. The degree of colitis‐associated cancer (CAC) formation was observed by high resolution mini‐endoscopy (STOKE, Germany) and then, the mice were killed at 9 weeks post‐AOM injection.^16^ After this, whole colons were removed and flushed with phospate‐buffered saline and portions of the distal colons were either frozen in liquid nitrogen for total RNA extraction or fixed with 4% formalin and paraffin‐embedded for histological analyses.

### 2.3 Histopathology

Paraffin‐embedded colon tissues were cut into 4 μm slides. After deparaffinization with xylene and hydration with gradient alcohol (100%, 95%, 90%, 80%, 70% for 10 minutes), the slides were sequentially stained with haematoxylin and eosin (H&E; Beyotime, Beijing, China). After mounting with neutral balsam, the slides were photographed using an Olympus BX51 upright microscope (Tokyo, Japan).

### 2.4 Total RNA extraction

Total RNA was extracted from colon tissues using TRIzol reagent (Invitrogen, MA, USA), following the manufacturer's instructions. The concentration of total RNA was quantified by the NanoDrop ND‐2000 (Thermo Scientific, MA, USA) and the RNA integrity was assessed using Agilent Bioanalyser 2100 (Agilent Technologies, Palo Alto, California, USA). The RNA with 28S/18S > 0.7 and 2100 RIN > 7.0 was used for further microarray analysis.

### 2.5 Microarray analysis

Sample labelling, microarray hybridization and washing were performed by Shanghai OE Biotech Co., Ltd. (Shanghai, China). Briefly, total RNA was transcribed to double stranded cDNA, then synthesized into cRNA and labelled with Cyanine‐3‐CTP. The labelled cRNAs were hybridized onto the microarray Mouse lncRNA V3 (4*180K, Design ID:084388; Agilent Technologies). After washing, the arrays were scanned by the Agilent Scanner G2505C (Agilent Technologies). Feature extraction software (version 10.7.1.1; Agilent Technologies) was used to analyse array images to get raw data. Genespring (version 14.8; Agilent Technologies) was employed to complete the basic analysis using the raw data. First, the raw data were normalized with the quantile algorithm. The probes that met at least 1 of 2 conditions with flags in ‘P’ were chosen for further data analysis. Differentially expressed genes or lncRNAs were then identified through fold change as well as *P* values calculated with a *t* test. The threshold set for up‐ and down‐regulated genes was a fold change ≥2.0 and a unadjusted *P* value ≤0.05. Three biological replicates are included at each time‐point in the microarray analysis.

### 2.6 Quantitative PCR analysis

TRIzol reagent (Invitrogen) was used to isolate total RNA from mouse intestinal tissues and then the PrimeScript RT Reagent Kit (Perfect Real Time, Takara, Tokyo, Japan) was used for reverse transcription, following the manufacturer's instructions. Using TB Green Premix Ex TaqTM II (Tli RNaseH Plus, Takara, Tokyo, Japan), cDNA samples were amplified to set up a standard curve for calculating the relative expression of RNAs. In addition, we used a housekeeping gene, β‐actin, as an internal reference. The sequences of primers used for quantitative real‐time PCR (qPCR) are listed in as in Table 1. The reaction of qPCR was set at 95°C (30 seconds) for pre‐denaturation, then 95°C (5 seconds) and 58°C (30 seconds) for a total of 42 cycles. A final dissociation curve was obtained.

**Table 1:** Sequences of primers used for real‐time PCR

Primer names	Sequences
Scgn sense	5′‐ATCAGTGGTGTGGATCTGGA‐3′
Scgn anti‐sense	5′‐TTTCCATCCTTGTTGACATCGC‐3′
Wif1 sense	5′‐AAGCAAGTGTAAGTGCCCGA‐3′
Wif1 anti‐sense	5′‐AGGCTGGCTCCATACCTCTT‐3′
mmp7 sense	5′‐AGCGCACATCAGTGGGAACA‐3′
mmp7 anti‐sense	5′‐TGTAGGGGGAGAGTTTTCCAGT‐3′
XR_373414 sense	5′‐CTGGATGGTTTCGAGGATCA‐3′
XR_373414 anti‐sense	5′‐CTCTACAGGAACTACAGAAGG‐3′
BC066854 sense	5′‐AATGGATAAAGACCAGTGTGC‐3′
BC066854 anti‐sense	5′‐ATGAAGTAGAGAGAACAAGACG‐3′
AK195973 sense	5′‐GCAGATACTCAACAGCCTTC‐3′
AK195973 anti‐sense	5′‐CCATCCGTGAGCTGATTTC‐3′
H19 sense	5′‐GATGACAGGTGTGGTCAATG‐3′
H19 anti‐sense	5′‐CTGCCCTTCTGTCCTCTC‐3′

### 2.7 *K*‐means analysis

*K*‐means clustering of unions of the differential expression genes (DEGs) was performed using R package.^17^ based on the fuzzy *K*‐means algorithm. The expression of genes within the same cluster was similar and may have common function. Total 6‐15 *K*‐means clusters were divided based on the gene expression values and *K*‐means = 12 was selected as the optimal one. Enrichment analysis was conducted on every cluster of genes.

### 2.8 Weighted correlation network analysis

LncRNA and mRNA co‐expression networks were constructed using the weighted correlation network analysis (WGCNA) package in R language^18^ and Cytoscape were used for data visualization. The gene co‐expression network is a scale‐free weighted gene network. A significant feature of scale‐free networks is that most nodes have only a few connections, with a few nodes having a large number of connections. To satisfy the precondition of scale‐free network distribution, the adjacency matrix weight parameter β value needed to be determined. In this study, we evaluated β values from 1 to 20, and the corresponding correlation coefficient and mean value of the adjacent gene were calculated for each. A higher correlation coefficient (maximum = 1) indicates that the network is closer to the network size distribution. At a certain degree of gene connectivity, the β value should be as small as possible when the correlation coefficient is sufficiently large. Therefore, we selected β = 11 to construct the co‐expression networks. Based on the above analysis, we constructed a WGCNA to subdivide thousands of genes into several modules. To describe the most common model of gene expression in each module, we conducted singular value decomposition of the gene expression values in every module and obtained multiple singular values and their corresponding eigenvectors. The characteristic vector with the highest degree of variation in gene expression in each module was defined as the characteristic gene expression of the module. We extracted the featured genes of each module for all samples and then calculated their association with the time‐point traits. We drew heat maps for each module based on correlation coefficients, with a deeper colour representing a higher degree of correlation. To further explore interactions among genes in each module, we selected those genes with the highest connectivity to draw the gene network. In addition, information regarding the functions of differentially expressed genes was collected from unigene annotations. These genes were subjected to gene ontology (GO) significant enrichment analyses to identify the biological functions and metabolic pathways in which these genes participate. In network biology analysis, hub genes are good representatives of each module relative to other genes in the module and have important biological significance in system analysis.

### 2.9 TCGA analysis

Read counts of both mRNA and lncRNA in colon adenocarcinoma tissue samples were downloaded from Genomic Data Commons (GDC, https://portal.gdc.cancer.gov). Differential expression analysis was performed to identify DEGs using the DESeq pipeline. Significant DEGs were defined as genes with abs(LogFC) ≥ 1 and adjusted *P* value <0.01. Normalized expression values of lncRNAs in normal tissue and tumour tissue were visualized as boxplots.

### 2.10 Statistical analysis

Numerical continuous data were presented as the mean ± standard deviation and were analysed using Student's *t* tests for comparing two groups and analysis of variance for multiple group comparisons. Statistical analysis was performed using SPSS version 19.0 (SPSS, Inc., Chicago, IL, USA). Kaplan‐Meier curves and the log‐rank test were used to compare survival times among the groups. *P* < 0.05 was considered to indicate a statistically significant difference.

## 3 RESULTS

### 3.1 Induction and identification of primary colon tumours in mice

**Figure 1:**
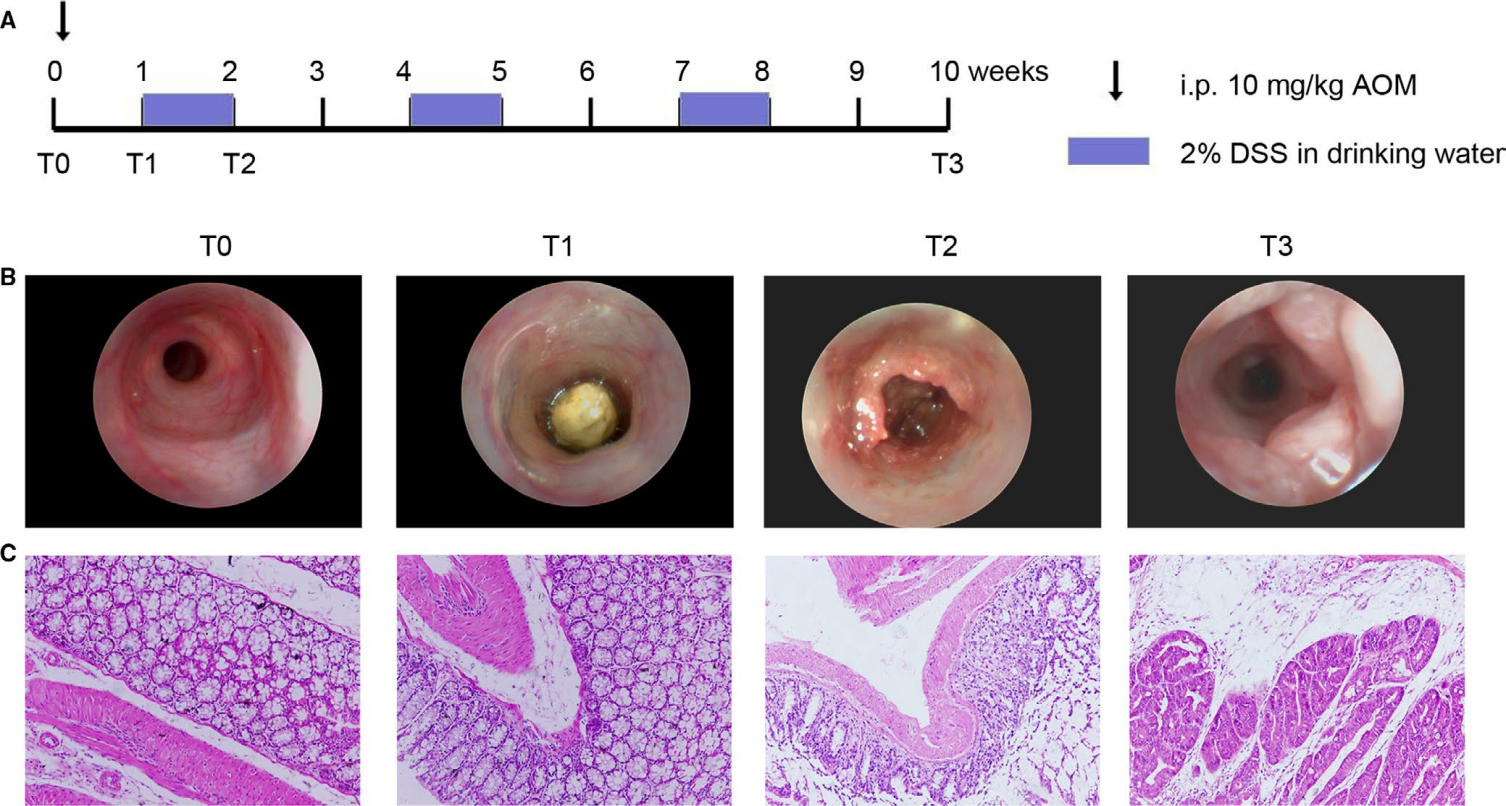
Establishment of primary colon cancer in mice. A, Schematic overview of the azoxymethane (AOM) and dextran sodium sulfate (DSS)‐induced colon cancer model in mice. A single AOM injection (10 mg/kg) is followed by three cycles of 2% DSS administration in the drinking water. The T0, T1, T2 and T3 labels indicated the time points of 0, 1, 2 and 10 wk post‐AOM injection. B, High resolution mini‐endoscopy images of colon cross‐sections at T0, T1, T2 and T3. Severe colitis was observed at T2 whereas colon tumours were detected at T3. C, Representative images of haematoxylin and eosin staining of colon tissues at T0, T1, T2 and T3. Transmural leucocyte infiltration and loss of goblet cells occurred at T2 while tumors were observed at T3. Scale bar = 100 μm

To investigate the expression and functions of deregulated lncRNAs in colon tumour initiation, the AOM/DSS induced primary colon cancer model was established (Figure 1A). Over the interval of a week, the colonoscopy was performed and colon tissues were collected. The colonoscopy indicated severe colitis at 2 weeks post‐AOM injection (named as T2 time‐point) while solid colon tumours were detected at 10 weeks post‐AOM injection (named as T3 time‐point) (Figure 1B). H&E staining confirmed severe inflammation at T2, as indicated by transmural leucocyte infiltration and loss of goblet cells (Figure 1C) and an apparent tumour area in colon tissues at T3 (Figure 1C). Collectively, we induced and identified a primary colon cancer model in mice that mimics the development of colon cancer in human patients.

### 3.2 Deregulation of lncRNA expression in colon tumour initiation

**Figure 2:**
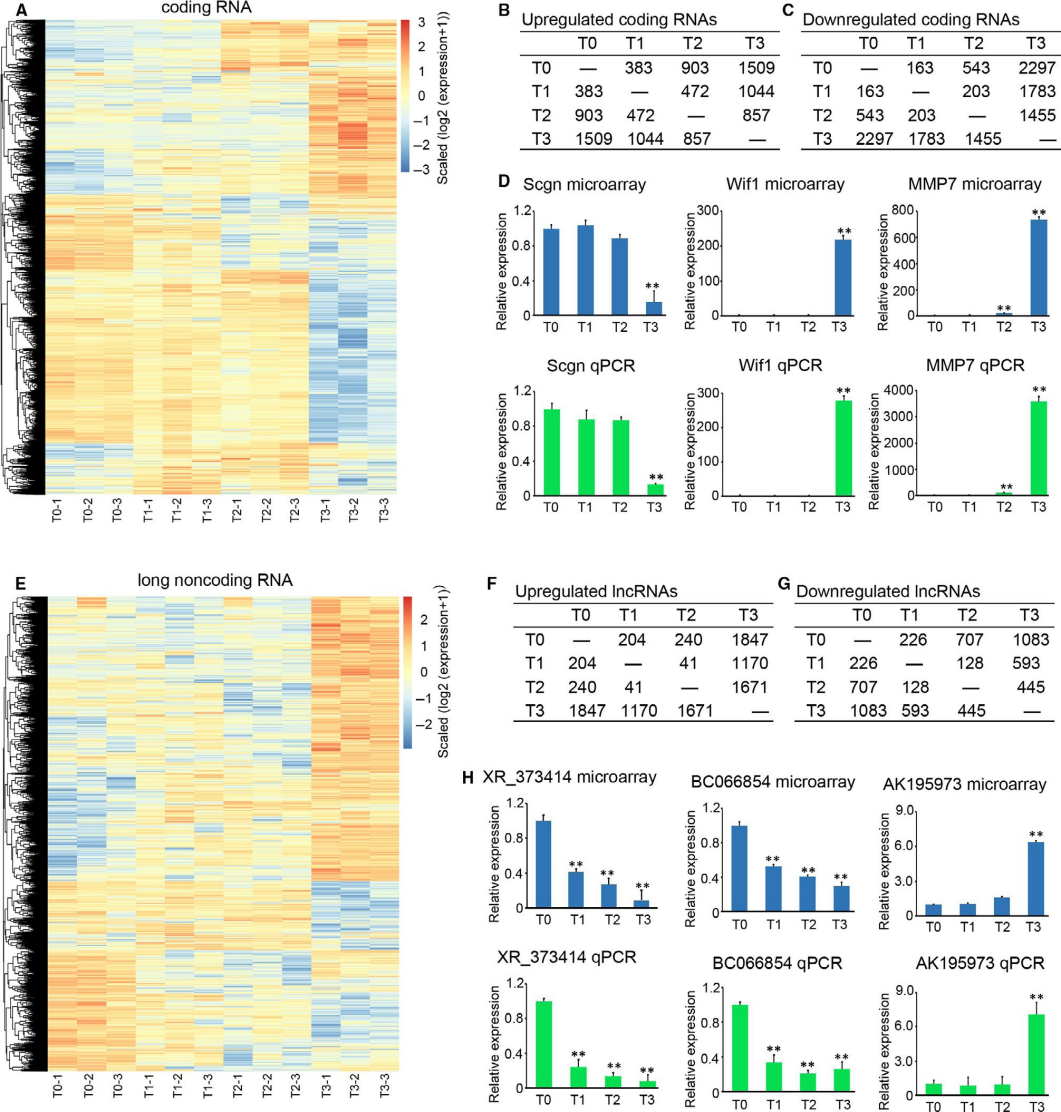
Differential expression of coding RNAs and long non‐coding RNAs (lncRNAs) during colon cancer formation. A, Heatmap of differential expression of coding genes during colorectal cancer (CRC) formation. B, The list and number of up‐regulated coding genes at T0, T1, T2 and T3. C, The list and number of down‐regulated coding genes at T0, T1, T2 and T3. D, Three random coding RNAs (Scgn, Wif1 and MMP7) were selected for qPCR determination. The barplot in the upper panel is the expression pattern of genes determined by microarray. The barplot in the lower panel is the expression pattern of genes determined by qPCR (n = 3, **P < 0.01, compared with T0). E, Heatmap of differential expression of long non‐coding genes during CRC formation. F, The list and number of up‐regulated long non‐coding genes at T0, T1, T2 and T3. G, The list and number of down‐regulated long non‐coding genes at T0, T1, T2 and T3. H, Three random coding lncRNAs (lncRNAs XR_373414, BC066854 and AK195973) were selected for qPCR determination. The barplot in the upper panel is the expression pattern of lncRNAs determined by microarray. The barplot in the lower panel is the expression pattern of lncRNAs determined by qPCR (n = 3, **P < 0.01, compared with T0)

Next, colon tissues of T0, T1, T2 and T3 were collected for microarray determination. The results suggested that 546 coding RNAs (383 up‐regulated and 163 down‐regulated) were deregulated at T1, 1446 coding RNAs (903 up‐regulated and 543 down‐regulated) at T2 and 3806 (1509 up‐regulated and 2297 down‐regulated) coding RNAs at T3 compared with T0, while 675 coding RNAs were deregulated at T2 and 2827 coding RNAs were deregulated at T3 compared with T1 (Figure 2A,B). Furthermore, at T3, 2312 coding RNAs were deregulated compared with T2 (Figure 2A,B). To confirm the accuracy of the microarray, three random coding RNAs (Scgn, Wif1 and MMP7) were selected for qPCR determination. We demonstrated that the expression trends of Scgn, Wif1 and MMP7 detected by qPCR were confirmed with the results from microarray (Figure 2C). Microarray results also suggested that 430 lncRNAs (204 up‐regulated and 226 down‐regulated) were deregulated at T1, 947 lncRNAs (240 up‐regulated and 707 down‐regulated) at T2 and 2930 lncRNAs (1847 up‐regulated and 1083 down‐regulated) at T3 compared with T0, while 169 lncRNAs were deregulated at T2 and 1763 lncRNAs were deregulated at T3 compared with T1 (Figure 2D,E). Furthermore, 2116 deregulated lncRNAs were determined at T3 compared with T2 (Figure 2D,E). Three random lncRNAs, XR_373414, BC066854 and AK195973 were also selected for qPCR analysis. The results in Figure 2F indicated that the expression trends of XR_373414, BC066854 and AK195973 confirmed the results from microarray. The above results identified the deregulated lncRNAs and coding RNAs in colon tumour initiation.

### 3.3 Time‐course analysis of lncRNAs expression patterns in colon tumour initiation

**Figure 3:**
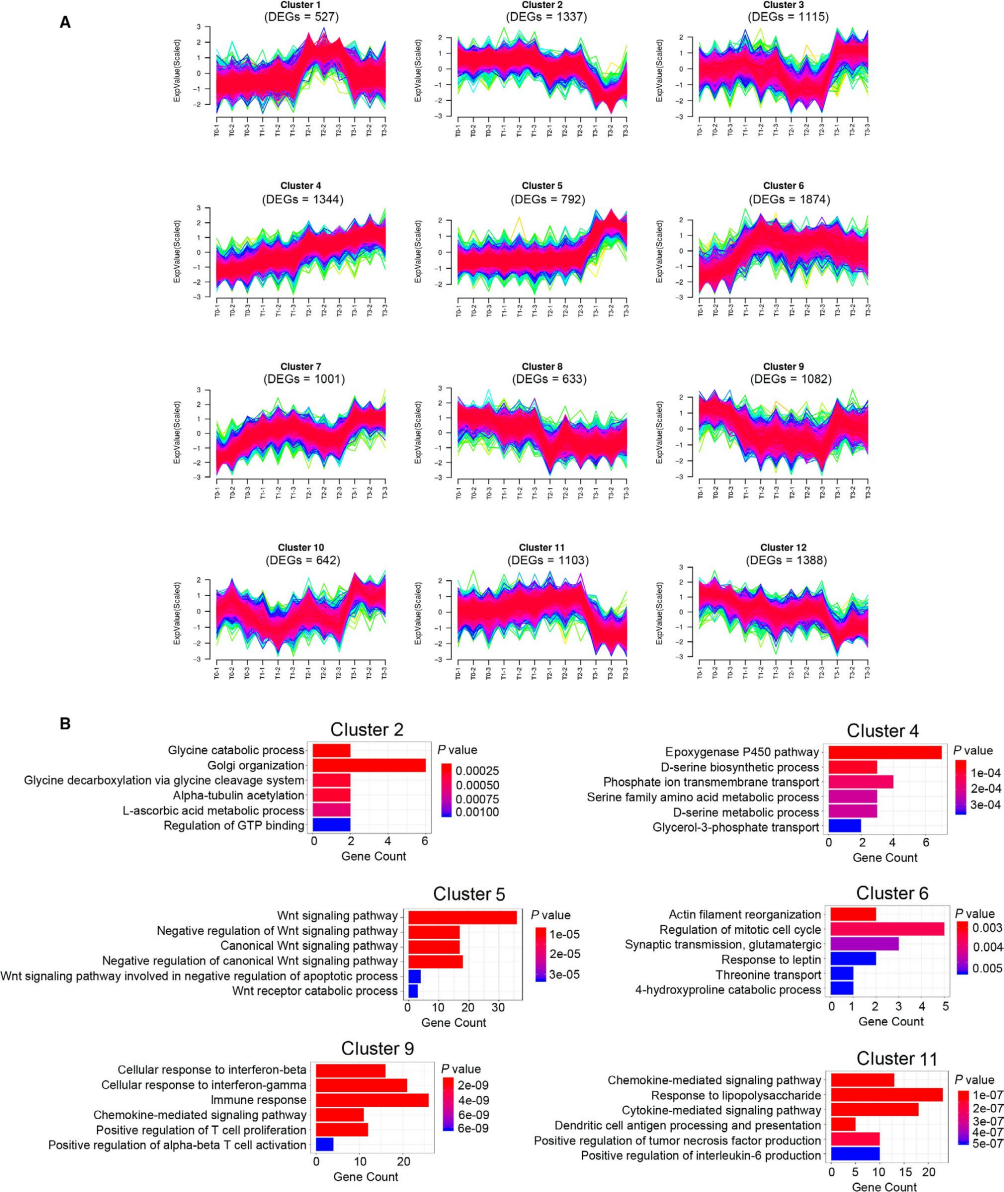
Temporal expression analysis of deregulated long non‐coding RNAs and coding RNAs. A, *K*‐means clustering of unions of the differential expression gene (*K* = 12). Each line indicates one differential expression gene. The red line indicates the high similar one and the green line indicated the low similar one. B, Gene ontology biological process term enrichment of the four *K*‐means clusters. The top six results are plotted

To investigate the temporal expression of DEGs, the common 4307 differential expressed lncRNAs and 5798 differential expressed mRNAs expression values were used as input data to analyse patterns in gene expression by *K*‐means analysis. The expression of gene within the same cluster was similarly and may have common function and 12 *K*‐means clusters were defined. As shown in Figure 3A, 1344 DEGs in cluster 4 were gradually raised and kept at a high level at T3. Meanwhile, 1874 DEGs in cluster 6 were raised at T1 and stabilized at a high level from T1 to T3 (Figure 3A), whereas 792 DEGs in cluster 5 were kept at a base level from T0 to T2 and only raised at T3 (Figure 3A). Moreover, DEGs in cluster 2 (1337 DEGs), 9 (1082 DEGs) and 11 (1103 DEGs) reduced at T2, T1 and T3 separately (Figure 3A). To further understanding the potential functional role of DEGs with obvious temporal correlation, GO‐BP analysis of cluster 2, 4, 5, 6, 9 and 11 were performed. As shown in Figure 3B, Wnt signalling pathway was enriched in cluster 5, which only raised at T3, while immune‐related processes and chemokine/cytokine expression‐related processes were enriched in cluster 9 and 11 separately. Collectively, these results suggested that the DEGs that have similar expression trends may play analogical functional role in CRC formation.

### 3.4 Weighted correlation network analysis of DEGs

**Figure 4:**
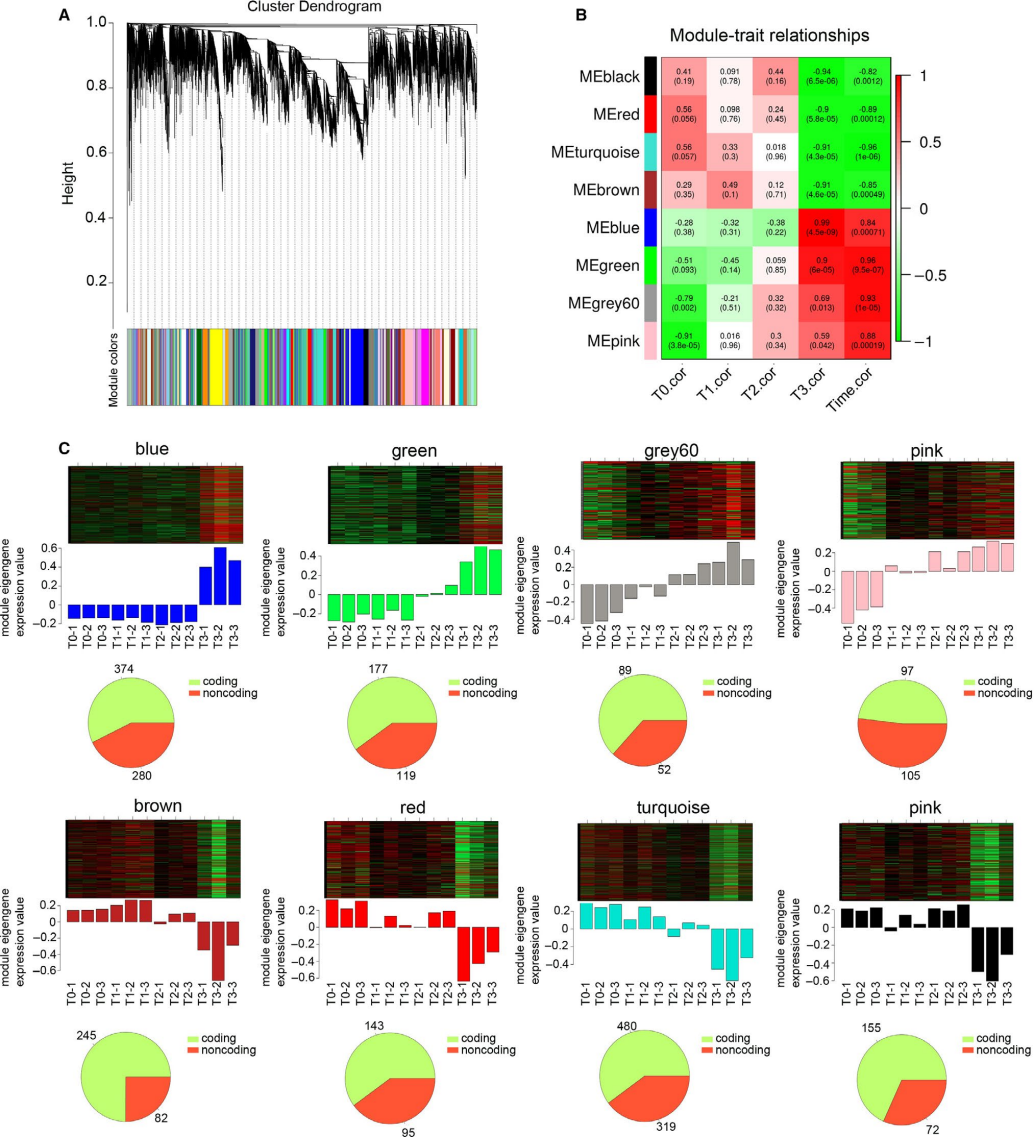
Weighted gene co‐expression network analysis (WGCNA) of deregulated long non‐coding RNAs (lncRNAs) and coding RNAs. A, Gene cluster dendrogram of deregulated lncRNAs and coding RNAs at T0, T1, T2 and T3. B, Stage specific co‐expression gene modules and their correlation to colon tumor formation. Numbers on each square represent the correlation between module and colon cancer stage and *P*‐value of each correlation value. The right column labeled ‘time correlation’ represents the correlation of each module and the entire development process of tumour initiation. Red square indicates a positive correlation, green square indicates a negative correlation and white square indicates no correlation. The *r* value and *P* value were included in each square. C, Heatmap in the upper panel is the expression pattern of all genes in the module across all 12 samples. The barplot in the middle panel shows the corresponding module gene expression value. The pie chart in the lower panel is the exact number of mRNAs and lncRNAs in each module

To identify genes expressed together as modules on a higher systems level, a WGCNA was carried out to associate lncRNAs with mRNAs and predict their functions. All DEGs were divided into 53 gene clusters based on the predicted functions (Figure 4A). To infer the dynamic biological process through time‐point samples, we also introduced temporal correlation as an important evaluation index for selecting the functional modules. As shown in Figure 4B, eight modules were highly correlated (time correlation > 0.8, *P *<* *0.01) with time throughout the entire process of CRC formation. The functional annotations enriched in each module indicated that they are clearly functionally related to specific stages of CRC formation (Figure 4C). In each stage, especially in CRC‐formatted stage, we observed a large number of up‐regulated and down‐regulated lncRNAs, suggesting lncRNAs might be involved in biological processes which regulate the initiation of CRC. Genes in the blue module, which is highly related to CRC formation, included up‐regulation of 374 protein‐coding RNAs and 280 lncRNAs, while in the brown module, 245 mRNAs and 82 lncRNAs were down‐regulated (Figure 4C). Notably, the genes in the green and grey60 modules were gradually up‐regulated correlating with the progress of colitis and CAC formation (Figure 4C, 177 mRNAs and 119 lncRNAs were gradually up‐regulated in the green module while 89 mRNAs and 52 lncRNAs were gradually up‐regulated in the grey60 module). These results suggested that lncRNAs in the blue, brown, green and grey60 modules regulated colitis and CAC formation.

### 3.5 Functions of lncRNAs in colon tumour initiation

**Figure 5:**
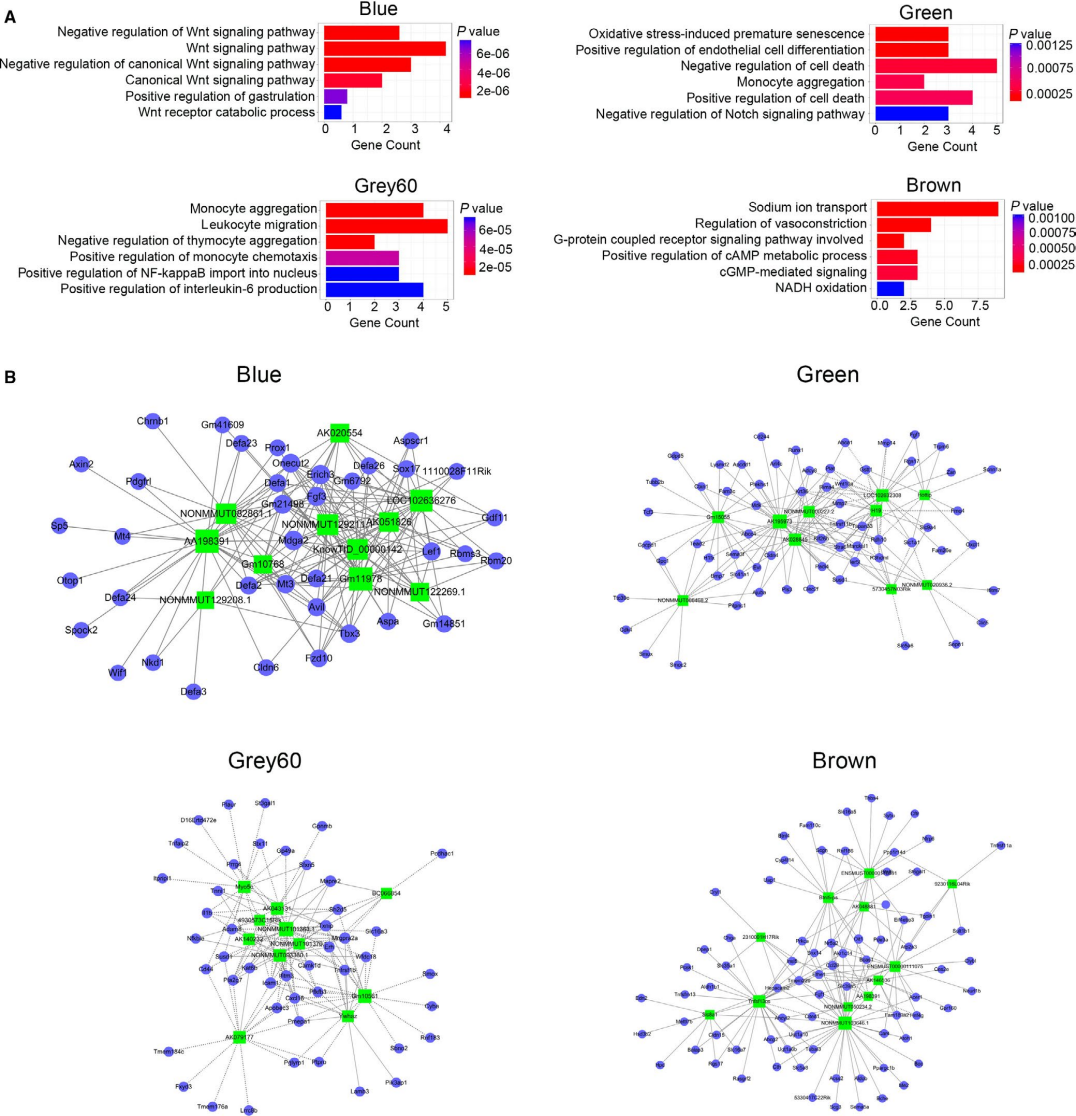
Function analysis deregulated long non‐coding RNAs (lncRNAs) and coding RNAs. A, Gene ontology biological process term enrichment of the four modules (blue, green, grey60 and brown), top six results are plotted. B, Co‐expression connections of lncRNAs and coding RNAs in the blue, green, grey60 and brown modules. The full line indicates a positive correlation, and the dotted line indicates a negative correlation

Next, these genes in blue, brown, green and grey60 modules were subjected to GO significant enrichment analyses to identify the biological functions and metabolic pathways in which these modules participate. Biological process analysis showed that the blue module was enriched with the Wnt signalling pathway, which plays a crucial role in regulating stemness of cells and promoting cancer formation (Figure 5A). Meanwhile, the cell death regulation pathway and Notch signalling pathway were both involved in green module (Figure 5A). Genes in the grey60 module were gradually up‐regulated at the specific stage of colon inflammation appearance. Additionally, biological process analysis indicated that immune‐related processes (monocyte aggregation, leucocyte migration, negative regulation of thymocyte aggregation, positive regulation of monocyte chemotaxis, positive regulation of NF‐κB import into nucleus and positive regulation of IL‐6 production) are enriched associated with the colitis phenotype (Figure 5A). Furthermore, G‐protein coupled receptor‐related signalling was associated with the function of these genes in brown (Figure 5A). To define the hub genes during CRC formation, lncRNA‐mRNA co‐expression in the above four modules was established and the lncRNAs that associated with more than five mRNAs were defined as hub genes (Figure 5B). The defined 11 hub lncRNAs in the blue module were: AK020554, LOC102636276, NONMMUTO082861.1, AA198391, NONMMUT129211.1, AK051826, Gm10768, KnowTID_00000142, NONMMUT129208.1, Gm11978 and NONMMUT122269.1 (Figure 5B). Furthermore, Myo50, AK043134, BC066854, 4903573C15Rlk, NONMMUT1013363.1, AK140232, NONMMUT101370, NONMMUT093360.1, Gm10561, AK079177 and Ywhaz were defined as the hub lncRNAs in the grey60 module (Figure 5B). Meanwhile, ten hub lncRNAs and 12 hub lncRNAs were defined in the green and brown modules, respectively (Figure 5B). These results shown that deregulated lncRNAs function in central regulatory roles in CAC formation.

### 3.6 LncRNA H19 predicts the prognosis of colon cancer patients

**Figure 6:**
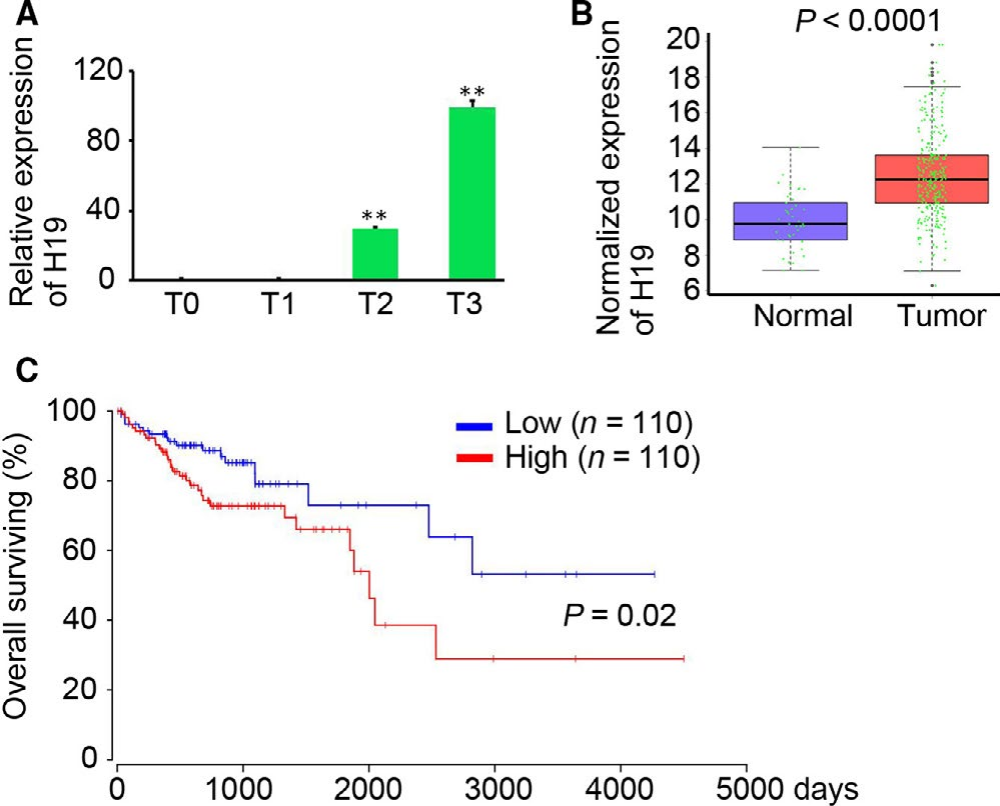
Long non‐coding RNAs (lncRNAs) H19 predicts poor prognosis of colon cancer patients. A, The relative expression of H19 at T0, T1, T2 and T3 (n = 3, **P < 0.01, compared with T0 group). B, The normalized expression of H19 in colon cancer tissues and normal colon tissues. The read counts of lncRNA H19 of colon tissue samples (41 normal tissue samples and 285 tumour tissue samples) were download from Genomic Data Commons (https://portal.gdc.cancer.gov), and normalized expression was performed. C, Prognosis analysis of H19 high expression (n = 110) and low expression (n = 110) colon cancer patients (*P* = 0.02)

LncRNA H19 has been demonstrated to be an oncogene and is up‐regulated in several cancers.^19^, ^20^, ^21^ We also demonstrated that H19 expression was gradually increased along the colon cancer initiation in mice (Figure 6A). The read count data of normal colon tissues (n = 41) and colon tumour tissues (n = 285) downloaded from the GDC database also indicated the up‐regulation of H19 expression in malignant colon tissues (Figure 6B). Furthermore, the 220 colon patients were divided into the H19 high expression group and the H19 low expression group according to the median expression of H19. The prognosis analysis suggested that high H19 expression predicted poor prognosis of colon patients (Figure 6C). Collectively, up‐regulation of H19 expression negatively correlated with prognosis of colon patients.

## 4 DISCUSSION

Recent studies have demonstrated that lncRNAs are great biomarkers in clinical diagnosis and are potential targets in therapies for various cancers.^22^ In this study, we first determined the expression profile of lncRNAs during CRC formation based on a CAC model in mice. Further analysis demonstrated the temporal expression and predicted function of lncRNAs associated with the development of CRC. A series of lncRNAs were identified as hub genes in regulating cell stemness, colon inflammation, oxidative stress response and cell death at each specific stage.

Various studies have aimed to explore the expression profile of mRNAs, miRNAs and lncRNAs in CRC and develop novel diagnosis and classification biomarkers.^23^, ^24^, ^25^, ^26^, ^27^ A previous study by Linza has indicated the differences in miRNA expressions, specifically between microsatellite stability (MSS) versus high microsatellite instability (MSI‐H) CRCs and that the combination of mRNA/miRNA expression signatures could be used to improve biomolecular classification of human cancers.^27^ Differential expression of lncRNAs between CRC tissues and normal tissues was also demonstrated and the results suggested that more than 1000 lncRNAs were differentially expressed in CRC tissues compared with paired normal adjacent tissues.^25^, ^28^ However, no study was performed to investigate the expression profile of lncRNAs throughout CRC initiation. In this study, we first demonstrated the temporal expression of lncRNAs in CRC initiation based on an AOM/DSS‐induced CAC model. The results suggested that 4307 lncRNAs were deregulated during CRC formation, and these lncRNAs presented differential expression trends demonstrated by *K*‐means clustering analysis. Notably, the different *K*‐means cluster that have similar expression trends play different functional roles in CRC formation. Thus, understanding the temporal expression of lncRNAs during CRC formation would be better predicting the function of the specific lncRNAs.

Aberrant activation of Wnt signalling pathway plays a role in promoting colon carcinogenesis. As our results indicated, the Wnt signalling pathway was located in the blue module that increased only at the colon cancer formation stage. Co‐expression analysis showed that 11 hub lncRNAs were involved in the blue module, including Gm10768, which has been demonstrated to activate hepatic gluconeogenesis.^29^ Both Frizzled homolog 10 (FZD10), which has a distinct role from other FZDs in canonical Wnt signal transduction,^30^ and Mt3, which was activated through beta‐catenin dysregulation,^31^ were positively correlated with the expression of Gm10768. This further predicts the functional role of Gm10768 in the Wnt signaling pathway, but additional experiments should be performed to demonstrate the exact function of Gm10768, as well as other lncRNAs included in blue module.

In the AOM/DSS‐induced CAC model, AOM functioned as a mutation inducer while DSS caused epithelial damage and subsequently, colon inflammation. Our WGCNA results indicated that 52 lncRNAs and 89 mRNAs in the grey60 module were up‐regulated at the T2 time‐point (1 week post‐DSS adding in drinking water). GO‐BP analysis suggested that these genes were involved in monocyte aggregation, leucocyte migration and related pathway (NF‐kappaB, IL‐6), which play a central role in colon inflammation.^32^, ^33^, ^34^ Thus, we predicted that these lncRNAs Myo50, AK043134, BC066854, 4903573C15Rlk, NONMMUT1013363.1, AK140232, NONMMUT101370, NONMMUT093360.1, Gm10561, AK079177 and Ywhaz may play a functional role in colon inflammation, even for CAC.

Higher expression of lncRNA H19 was observed in colon cancer and significantly correlated with tumour differentiation and poor overall survival and poor disease‐free survival of CRC patients.^35^ A recent study by Geng et al also demonstrated increased H19 in inflamed intestinal tissues from mice and patients.^19^ Our results indicated that H19 was up‐regulated in inflamed colon tissues and CRC tissues. Further analysis based on the GDC data also demonstrated that H19 expression correlated with poor overall survival of CRC patients. The potential predicational role H19 expression for the survival of CRC patients should be determined in various cancers through a more larger database, such as TCGA data. Meanwhile, functional studies suggested that H19 up‐regulates VEGF to enhance mesenchymal stem cells survival and angiogenic capacity by inhibiting miR‐199a‐5p.^20^ Meanwhile, H19 was required for intestinal epithelial proliferation, mucosal healing via binding to p53 and microRNAs,^19^ and tendon healing through targeting miR‐29b‐3p.^36^ Notably, co‐expression analysis in our study suggested that H19 was positively correlated with Wnt10a and MMP7 expression, which play a central role in maintaining cell stemness and tissue repair.^37^, ^38^, ^39^ The identified hub lncRNAs in our study may play an important role in regulating various biological processes, but further experimental studies should be performed.

This study first clarified the temporal expression and function of lncRNAs at specific stages of CRC initiation. The defined hub lncRNAs that are involved in regulating cell stemness, colon inflammation, oxidative stress responses and cell death would be a series of novel and efficient diagnostic and therapeutic targets for CRC. Of course, due to the small number of samples, there do exist some limitations in the reported analysis. And further experimental studies should be performed to investigate the functions and underlying mechanism of these hub lncRNAs in CRC initiation.

## CONFLICT OF INTEREST

The authors declare that they have no potential conflict of interest.

## AUTHOR CONTRIBUTIONS

Lei Dai, Junshu Li, Zhexu Dong, Yi Liu, Ye Chen and Na Chen were involved in acquisition and analysis of the data. Hongxin Deng and Lei Dai were involved in the study concept and design and obtained funding. Lin Cheng, Chao Fang and Huiling Wang were involved in statistical analysis. Yi Lin, Yanhong Ji, Shuang Chen, Xiaolan Su and Gang Shi were involved in technical support for model establishment. Shuang Zhang and Yang Yang were involved in interpretation of the data. Meng Qiu, Dechao Yu and Wei Huang were involved in critical revision of the manuscript for important intellectual content. Zongguang Zhou, Yuquan Wei and Hongxin Deng were involved in study supervision.
